# Development of a Coaching System for Functional Electrical Stimulation Rowing: A Feasibility Study in Able-Bodied Individuals

**DOI:** 10.3390/s22051813

**Published:** 2022-02-25

**Authors:** Shirin Tajali, Kai Lon Fok, Pirashanth Theventhiran, Gongkai Ye, Hikaru Yokoyama, Kento Nakagawa, Kei Masani

**Affiliations:** 1 KITE Research Institute, Toronto Rehabilitation Institute—University Health Network, Toronto, ON M4P 1E4, Canada; Canadashirin.tajalli@utoronto.ca (S.T.); kailon.fok@mail.utoronto.ca (K.L.F.); pirashanth.theventhiran@mail.utoronto.ca (P.T.); jerry.ye@mail.utoronto.ca (G.Y.); yokoyama@idaten.c.u-tokyo.ac.jp (H.Y.); nakagawa.kento.22@gmail.com (K.N.); 2 Institute of Biomedical Engineering, University of Toronto, 164 College Street, Toronto, ON M5S 3G9, Canada; 3 Graduate School of Arts and Sciences, The University of Tokyo, 3-8-1 Komaba, Meguro-ku, Tokyo 153-8902, Japan; 4 Faculty of Sport Sciences, Waseda University, Saitama 359-1192, Japan

**Keywords:** functional electrical stimulation, rowing, rehabilitation, coaching, system development

## Abstract

Background: Functional electrical stimulation (FES) during rowing has substantial effects on cardiovascular health in individuals with spinal cord injuries. Currently, manual stimulation control where stimulation is operated by rowers is mostly utilized. However, it takes time to obtain the skill to initiate FES at the optimal timing. The purpose of this study was to develop a coaching system that helps rowers to initiate FES at the optimal timing. Methods: The optimal range for FES application was identified based on the electromyography of the left quadriceps in 10 able-bodied individuals (AB). Then, the effects of the coaching system on the timing of button-pressing, power, and work were investigated in 7 AB. Results: Vastus lateralis (VL) activation began consistently before the seat reached the anterior-most position. Therefore, seat position at the onset of VL was used as the variable to control the switch timing in the coaching system. The results revealed significantly higher power and work outputs in the coaching than the no-coaching condition (median power coaching: 19.10 W, power no-coaching: 16.48 W, *p* = 0.031; median work coaching: 109.74 J, work no-coaching: 65.25 J, *p* = 0.047). Conclusions: The coaching system can provide the optimal timing for FES, resulting in improved performance.

## 1. Introduction

Spinal cord injuries (SCI) affect around 500,000 individuals per year around the world [1]. These individuals often experience a deterioration in their cardiovascular and musculoskeletal systems due to physical inactivity after injury [1,2,3]. Increasing evidence indicates that aerobic exercises with moderate to vigorous intensity are greatly beneficial for these individuals [4]. However, due to muscle paralysis, exercises are typically restricted to the upper body, such as arm crank ergometry and weight training [5,6]. Notably, these exercises have limited ability to produce the high exercise intensity required for cardiovascular adaptation, as only a small amount of muscle mass is typically involved [3,7].

One possible solution to increase exercise intensity and enhance cardiovascular performance is through functional electrical stimulation (FES) assisted exercise [7,8,9]. FES-leg exercises can activate a larger amount of muscle mass by stimulating the major lower limb muscles [7]. As a result, the exercise intensity is significantly higher when compared with traditional arms-only exercises [7]. Recently, hybrid FES exercises which combine voluntary upper limb motion with FES have shown to induce greater aerobic demands, as determined by higher maximal oxygen consumption (VO_2_), than FES exercises alone [7,8,9]. The two popular forms of hybrid FES exercise are FES during cycling (FES-cycling) and FES during rowing (FES-rowing). In FES-rowing, the upper and lower limbs are engaged in a coordinated manner, leading to additional benefits, such as the activation of the lower limb muscle pump for venous return, and improvements in bone mineral density [3,7,9]. Also, VO_2_ during FES-rowing is found to be significantly higher than in arms-only or FES-cycling-only exercises [3,7,9,10,11].

In the FES-rowing exercise, cyclic flexion-extension of the knee is achieved by electrically stimulating the quadriceps and hamstrings muscles in the drive and recovery phases of a rowing cycle, respectively [8,9,11,12]. There is a wide variety of methods to administer stimulation during this exercise, including open-loop controllers (manual stimulators with push buttons) and closed-loop controllers (proportional derivative controllers, fuzzy logic controllers) [7,8,9,13,14,15]. In the open-loop systems, stimulation switching is typically controlled by the rower, i.e., pressing a button on the ergometer handle will result in stimulating the quadriceps and knee extension in a fixed pattern [8,16]. Additionally, releasing the button or pressing another button will lead to hamstring stimulation and knee flexion in another fixed pattern [8,16]. With regards to closed-loop controllers, stimulation is typically delivered automatically, based on the handle or seat position [13,14,17]. This type of control is usually preferred in patients who have difficulty with pushing the manual button due to a lack of voluntary control [14,18]. To date, most studies have relied on manual self-administration of FES, primarily because of rowers’ preference and comfort [3,8,9,11,15,16]. However, FES administration at the optimal time is not always an easy task. Indeed, this may take several sessions (approximately 13 ± 7) for individuals, especially those with SCI, to become accustomed to the movement and perform at a level with sufficient aerobic demand for reducing cardiovascular risks [9]. Furthermore, improper timing may impair coordination between upper and lower limbs, eventually preventing individuals from receiving the full benefits of this exercise [8]. Therefore, implementing a feedback system indicating the timing of FES administration, i.e., a coaching system, may accelerate the learning of button-press timing and enhance the training effects (musculoskeletal and cardiovascular benefits).

To date, there is no study investigating the effects of real-time audiovisual feedback on the optimal range for FES administration in the FES-rowing exercise. In this study, we have developed a coaching system that can help rowers to initiate FES-rowing at the optimal times. The ultimate goal of the coaching system is to improve the timing of FES administration in individuals with SCI. However, as a feasibility study, we initially determined the optimal range for FES application in able-bodied (AB) individuals during rowing on an instrumented rowing ergometer based on electromyography (EMG) activation of lower limb muscles. Then, we evaluated the effects of a coaching system on the FES-rowing performance. We hypothesized that the coaching system would help to improve the timing of manual button-pressing, which will be associated with higher work and power outputs.

## 2. Materials and Methods

This study consists of two experiments. In Study 1, we investigated quadriceps muscle activation during rowing with an instrumented rowing ergometer, and in Study 2, we developed a coaching system and investigated its effects on FES-rowing performance.

### 2.1. Participants

In total, 10 AB participated in Study 1 (1 female, age: 25.5 ± 3.75 years, height: 173.9 ± 7.1 cm and weight: 69.4 ± 11.6 kg), and 7 AB (1 female, age: 25.7 ± 3.50 years, height: 173 ± 6.25 cm, and weight: 69.7 ± 12.8 kg) participated in Study 2, while 7 participated in both studies. None of the participants reported any pain, neurological, or musculoskeletal injuries (e.g., back pain) at the time of experiments. Their rowing expertise ranged from individuals with no experience to one varsity rowing athlete. All participants signed an informed consent form detailing the experimental protocol, set up, and participated in both studies at Lyndhurst Rehabilitation Center. This study was approved by the Research Ethics Board of the University Health Network.

### 2.2. Modified Rowing Ergometer

**Figure 1:**
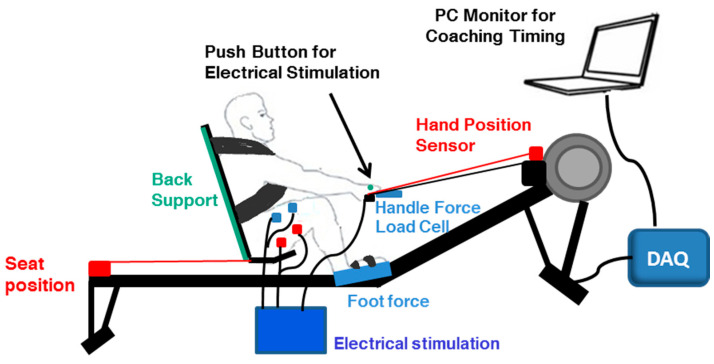
The instrumented functional electrical stimulation (FES) rowing machine with the coaching system.

We instrumented back and shank supports, shoulder straps, custom footplates, handle, and position sensors on a commercially available stationary rowing exercise machine (Concept2 model D, Concept2 Inc., Morrisville, VT, USA) (Figure 1). The back and shank supports, as well as the shoulder straps, were added to ensure the trunk and legs were held in a proper space. The position sensors were mounted to accurately capture the distance traveled by the handle and seat during rowing. For the handle position, the string potentiometer (Measurement Specialties, Durham Instruments, Scottsdale, AZ, USA) was mounted near the flywheel, so that the string was parallel to the handle chain. For seat position, the same string potentiometer was mounted at the posterior end of the ergometer and attached to the back of the seat using a small hook. The spring was adjusted for each individual to maximize the seat’s range of motion and to account for anthropomorphic differences in the thigh and shank length. Furthermore, the machine was instrumented with 5 load cells (GS1240-250 and SML-300, Interface Advanced Force Measurement Durham Instruments, Scottsdale, AZ, USA). Four load cells were mounted under the left and right footplates to measure both normal and shear forces along with the footplates, and one load cell was connected in series with the rowing handle. While this instrumented rowing ergometer was developed for various purposes in our lab, in Studies 1 and 2, we used only data related to the seat-position sensor. The analog signal was collected using a data acquisition system with a sampling frequency of 2000 Hz (PowerLab 16SP, ADInstruments Inc., Colorado Springs, CO, USA). The position data was filtered using a Butterworth low-pass filter with a cut-off frequency of 10 Hz.

### 2.3. Coaching System

The coaching system consisted of a laptop computer (Dell Inspiron 7559, Round Rock, TX, USA) and a data acquisition system (USB-6002 Multifunction I/O Device, National Instruments, South Portland, ME, USA) (Figure 1). The signal of the button-pressing and the position signal were collected in the coaching system via the data acquisition system at a sampling frequency of 200 Hz. We selected this sampling frequency as we found that previous studies in the biomechanics of rowing used frequencies around 100 Hz [8,19]. Therefore, we chose a higher sampling frequency to have more margin. The primary function of the coaching system was to provide real-time feedback and instruct the user on the optimal time to press the manual push button. The optimal timing for FES administration was defined based on the mean seat position at the onset of vastus lateralis (VL) activation in Study 1 and was input into the software written in Python on the laptop computer (Python Software Foundation, Python Language Reference, Version 3.5). Further details regarding methods for identifying the optimal timing are described in Section 2.4 and Section 3.1.

In terms of visual feedback during the coaching condition, seat position was shown in real-time, and the FES symbol turned yellow when the participant was within 2 standard deviations of the optimal range of seat positions so as to instruct participants to prepare to apply FES. Then, it turned green when the participant was within the optimal range so as to instruct participants to press the button and administer FES to their legs. This allowed the individual to anticipate FES timing and minimize their reaction-time delay. In terms of auditory feedback, a specific sound was emitted for correct button-pressing, and a different sound was emitted for the incorrect ones. Furthermore, feedback about the scores and misses were displayed, indicating correct and incorrect button-presses, respectively. With regard to pressing the button, there was a small button for FES application on the rowing handle, holding down the button stimulated the quadriceps, and releasing the button stimulated the hamstrings.

### 2.4. Experimental Procedures

#### 2.4.1. Study 1

In this study, we mainly focused on the EMG activation of knee extensor muscles including the VL, vastus medialis (VM) and rectus femoris (RF). Typically, in rowing, the drive phase is where most of the work is performed as one must work against the resistance of the water/handle, which was our target action. Furthermore, the return or recovery phase was assisted by a spring at the posterior end of the monorail and an inclined system in our study. Therefore, we assumed that the drive phase would be the most important phase of the rowing cycle with respect to the work output, and investigation of the optimal timing of button-pressing based on the EMG activation of the quadriceps may lead to a better design of the coaching system.

Each participant was set up with EMG electrodes on the left side of the body, including on the VL, VM, and RF. The center of each electrode was positioned at specific muscle locations, in agreement with the SENIAM recommendations [20]. The ground electrode was placed on the patella. The electrodes were taped and wrapped to the leg to prevent them from shifting during the rowing trials. These EMG signals were measured using an EMG amplifier (Bagnoli, Delsys Inc., Boston, MA, USA) and sampled at 2 kHz using a data acquisition system (PowerLab 16SP, ADInstruments Inc., Colorado Springs, CO, USA) synchronized with the other sensor signals mentioned above. We selected this sampling frequency based on our previous papers using EMG signals [21,22].

In the experiment, the participants performed 5 min of warm-up and familiarization on the instrumented rowing ergometer. They were instructed on the proper rowing technique prior to the initiation of the experiment. Then, they performed two minutes of rowing at the individual’s preferred speed while EMG activity of the lower limb muscles was collected. Thirty consecutive strokes in the middle of the 2 min rowing trials were used for the subsequent analyses.

#### 2.4.2. Study 2

Similar to Study 1, the participant performed 5 min of warm-up and familiarization with FES-rowing on the same rowing ergometer. Then, two rowing trials were performed, including FES-rowing trials (1) with the coaching system (coaching condition) and (2) without the coaching system (no-coaching condition). Each trial was randomly assigned and lasted for 2 min, followed by 5 min of rest. In each trial, the quadriceps and hamstrings were outfitted with adhesive gel electrodes (5 cm × 9 cm) for administering electrical stimulation. For quadriceps stimulation, the cathode was located on the muscle belly over the upper thigh, while the anode was placed distally just above the patella. For hamstring stimulation, the cathode was placed on the muscle belly over the proximal aspect of the posterior thigh, while the anode was placed distally above the popliteal fossa. A programmable 4-channel electrical stimulator (Compex Motion 2, Compex SA, Ecublens, Switzerland) was used to deliver rectangular, biphasic pulses to each target muscle. To determine the maximum tolerable intensity, it was fine-tuned for each participant, starting at 0 mA and increasing by 1 mA until the participants reported their maximum tolerable intensity. Then, 70% of the maximum tolerable intensity of each muscle was used in the FES-rowing trials. Based on the most common parameters typically used in FES-rowing studies, the stimulation parameters were 40 Hz frequency with 400 us pulse width [16].

Participants were asked to perform the FES-rowing trials in both conditions at their preferred speed. In the no-coaching condition, they were asked to operate the manual push button with an instruction to press the button once the seat reached the most-anterior position causing quadriceps stimulation, and to release the button once they reached the end of the rowing ergometer, resulting in hamstring stimulation. For the coaching condition, the participants were asked to follow the feedback on the coaching system to operate the manual push button. Thirty consecutive strokes in the middle of 2 min rowing trials were used for the subsequent analyses.

### 2.5. Data Analysis

#### 2.5.1. Study 1: Identifying the Seat Position at the Muscle Activation Onset

The EMG signals were de-meaned, full-wave rectified, and then filtered with a 4th order, low-pass Butterworth filter, and with a cut-off frequency of 10 Hz. To determine the onset of muscle activation for each rowing cycle, 10% of the EMG activity during the peak muscle activation in a rowing cycle was used as the threshold. Then, the seat position at the onset of muscle activation was determined for each cycle, and the mean and coefficient of variations (CVs) of the determined seat positions were calculated across 30 strokes for each muscle.

#### 2.5.2. Study 2: FES-Rowing Performance

The seat position at the time of button-pressing was measured for the 30 strokes. In addition, the number of strokes and the mean total external work and power were calculated for one minute in both the no-coaching and coaching conditions. Mean total external work was calculated as a function of the handle force and displacement in one minute and the mean power was calculated as a function of handle force by velocity in one minute.

### 2.6. Statistical Analysis

Firstly, the normality of each measure was tested using the Shapiro–Wilk test. For variables with normal distributions, parametric tests including paired *t*-tests and ANOVA were used, while for non-parametric variables, the Wilcoxon Signed Rank Test was used. Effect sizes were reported by Cohen’s d for paired *t*-tests, R-value for unpaired *t*-tests, and partial eta square (η^2^) for one-way ANOVA.

In Study 1, to determine which parts of the quadriceps muscle had the most consistent onset of activation relative to the seat position, the CVs for the seat position at the onset of VL, VM, and RF activations were compared using a one-way ANOVA (factor: muscle), and post-hoc analysis was performed using paired *t*-tests with a Bonferroni correction. Regarding the effect size, η^2^ values between 0.01–0.06, 0.06–0.25, and above 0.25 indicated small, medium, and large effects, respectively. Furthermore, to determine the effects of the coaching system on the timing of FES-administration, differences in the seat position at the time of button-pressing and also CVs of seat position were compared by using paired *t*-tests between the coaching and no-coaching conditions. Additionally, to determine the effects of coaching on the FES-rowing performance, differences in the total work and power outputs and stroke rates were compared by using many paired and unpaired *t*-tests between the 2 conditions. The level of significance for all tests was set to *p* < 0.05. A statistical software package (SPSS Statistics ver. 25, IBM Corp.,Armonk, NY, USA) was used for all statistical tests.

## 3. Results

### 3.1. Study 1

#### EMG Activation

**Figure 2:**
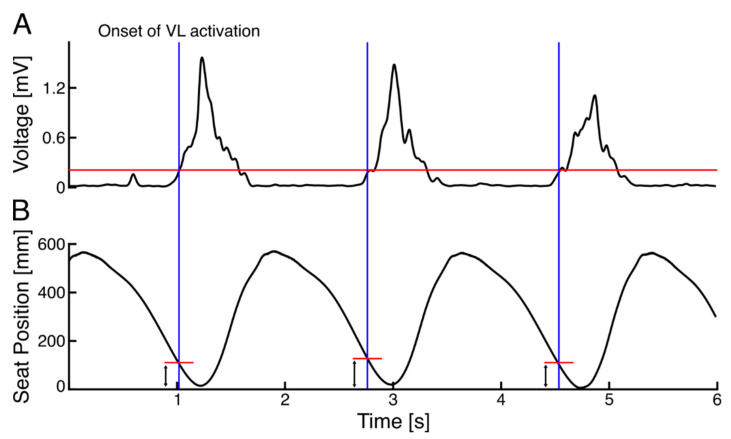
Example time courses of (**A**) vastus lateralis (VL) EMG envelope; and (**B**) inverted seat position signal across 3 rowing cycles from one participant. Red horizontal line in A indicates the threshold for VL onset detection and intersection of vertical blue line with the red horizontal line in B indicates the seat position at the onset of VL activation.

**Figure 3:**
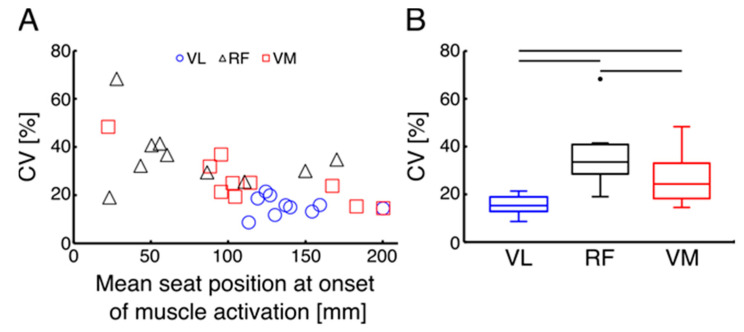
(**A**) Mean and coefficient of variations (CVs) of seat position at the onset of muscle activation across 30 consecutive strokes for ten subjects; (**B**) CVs of seat positions compared across muscles in ten subjects. Thin horizontal black lines at the top of the plot indicate a statistical significance (*p* < 0.05).

Figure 2 shows an example time course of VL activation and the seat position. It was found that the VL activation began slightly before the end of the rowing cycle, i.e., before the seat reached the anterior-most position. The seat position at the onset of each muscle was quantified and plotted in Figure 3A, showing the relation between the CV and the mean seat position at the onset of muscle activation across 30 strokes for each muscle. It shows that the CV for VL is clustering at the lower CV region around 100–150 mm of the seat position, while for the other muscles, the plots are scattering in wider regions. The results of the one-way ANOVA showed significant differences between muscles in terms of consistency in the onset of activation (*p* = 0.002, η^2^ = 0. 436). Further post-hoc comparisons in Figure 3B indicate that the CV for VL was significantly smaller than for the RF (*p* = 0.004) and for the VM (*p* = 0.043). Also, the CV for the VM was significantly smaller than for the RF (*p* = 0.021). This suggests that the VL activation is consistent against the seat position. As the group mean value of the seat position at the onset of VL was 140.5 ± 25.5 mm, we used 140 mm as the optimal timing of FES for the subsequent development of the coaching system.

### 3.2. Study 2

**Figure 4:**
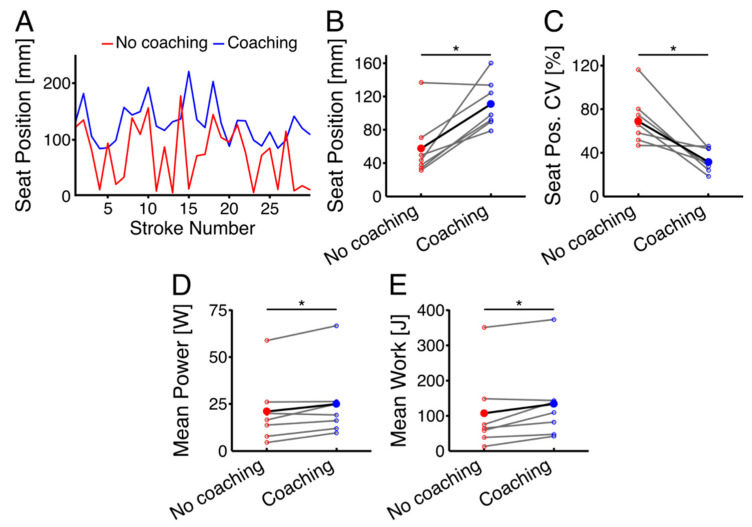
(**A**) Effect of coaching system on the seat position at the time of button-pressing across 30 strokes in a typical subject; (**B**) Seat position at the time of button-pressing in 7 able-bodied individuals for no-coaching and coaching conditions; (**C**) CV of the seat position in 7 able-bodied individuals for no-coaching and coaching conditions; (**D**) Mean total power; and (**E**) work outputs in the no-coaching and coaching conditions. Group means are shown as filled circles; individual participant data are shown as open circles. No-coaching condition is shown in red; coaching condition is shown in blue. Asterisks and horizontal black lines at the top of the figures indicate a statistical significance (*p* < 0.05).

Figure 4A–C represent the effect of the coaching system on the seat position at the time of button-pressing. The results of paired *t*-tests showed that there was a significant difference in the position of the seat at button-presses between the no-coaching vs. coaching conditions (*p* = 0.031, d = 1.465). Furthermore, there was a significant difference in the CV of the seat position at button-presses between the no-coaching and coaching conditions (*p* = 0.009, d = 1.450). A lower value indicates button-presses occurred closer to the anterior position. In the no-coaching condition, the seat position was much closer to the anterior-most position, indicating that participants were pressing the button as they reached the end of their motion. However, in the coaching condition, the button-pressing occurred consistently prior to reaching the anterior-most seat position.

Figure 4D,E show the results of FES-rowing performance, including work and power outputs, during the coaching and no-coaching conditions. The results of a Wilcoxon Signed Rank Test showed significantly higher power and work outputs in the coaching condition compared to the no-coaching condition, indicating an improved performance during FES-rowing exercise (improved time and increased distance rowed per stroke) (Power: *p* = 0.031, r = 0.542, Work: *p* = 0.047, r = 0.585). There was also a significant difference in the stroke rates per minute between the two conditions [no-coaching: 30.28 (7.60), coaching: 34.64 (7.20), *p* = 0.026, d = 0.588] indicating the relative time to complete the stroke length was shorter in the coaching condition.

## 4. Discussion

The first study was conducted to determine the optimal range for FES application based on quadriceps muscle activation during rowing on an instrumented rowing machine in the AB. The findings of our study confirmed that VL activation began consistently before the individuals reached the anterior-most seat position. Previous studies on FES-rowing with manual control switching relied mainly on the absolute seat position reaching the anterior-most position [8,9,12]. However, our results demonstrated that only analyzing absolute seat position may be insufficient in determining whether the targeted muscles are stimulated at the optimal time. Therefore, to guide the timing of the manual button-pressing for administering FES, seat position at the onset of VL activation was used. Concerning the coaching system, it appears to provide a safe method of improving FES-rowing performance as demonstrated by increased power and work during the coaching condition, indicating faster performance and longer distance rowed per stroke.

To better design the coaching system and to mimic leg muscle activation pattern during rowing, we investigated the EMG activation of the VM, VL, and RF as primary knee extensors in the AB. Our results indicate that activation of all parts of the quadriceps for knee extension occurred before the seat reached the anterior-most position. Specifically, VL activation was found to be more consistent across 30 consecutive strokes as determined by a significantly smaller CV of seat position at the onset of VL when compared with VM and RF. Similarly, in a study by Vieira et al. (2020) regarding lower limb muscle activation during indoor rowing, all parts of the quadriceps muscle were found to be recruited before the initiation of the drive phase to act as a braking mechanism and decelerate knee flexion at the end of a rowing cycle [12]. However, in the aforementioned study, consistency in the timing of muscle activation was not discussed among muscles [12]. In our study, calculation of a kinematic event (seat position) at the time of muscle activation showed that the VL can be an appropriate muscle to navigate the switch timing in the coaching system. Therefore, to guide the timing of the manual button-pressing for administering FES, seat position at the onset of VL activation was used.

To the authors’ knowledge, this is the first feasibility study that showed that a coaching system can result in the improvement of the timing of FES administration, leading to higher work and power outputs. It should be noted that the optimal seat position relative to the anterior-most position in Study 1 (around 140 mm) and the seat position at the time of button-pressing in Study 2 (around 110 mm) showed some delays around 30 mm, indicating that the participant required some response time to press the button. While the coaching system provided visual feedback to allow participants to anticipate the optimal timing and to minimize their reaction time delay, this information might not have been sufficient for participants to predict the timing perfectly. Further modifications may be required for the visual feedback to reduce the delay and to achieve FES administration at the optimal timing. Even though the delayed button-pressing resulted in about 30 mm of seat movement, using the coaching system still ensured that button-pressing occurred consistently before the seat reached the peak, as shown by a smaller CV of seat position in the coaching, compared to the no-coaching, condition. This is of great importance as, in the case of improper timing, the individuals may activate their leg muscles too late, and will thus rebound off the anterior stopper. In fact, the increased power and work seen during the coaching condition suggests that they were able to press the button more consistently within each stroke; and thus, the relative time to complete each stroke was shorter compared with the no-coaching condition. Regarding the importance of real-time feedback, Anderson et al. (2005) also showed that real-time visual feedback about actual kinematic data (shoulder and hip acceleration) can improve performance consistency during rowing in many experienced rowers when comparing summary feedback and no feedback conditions [23]. Another study comparing various types of multimodal feedback during simulated trunk-arm rowing in naïve subjects demonstrated that audiovisual feedback significantly improved learning of the velocity profile relative to visuo-haptic and visual feedback only [24]. Therefore, multi-modal audiovisual feedback received by individuals in our study regarding the timing of button-pressing and the scores of correct/incorrect button-pressing could have a high potential to enhance participant engagement and motivation during FES-rowing.

Although the preliminary findings of this study highlighted the immediate positive effects of the coaching system on FES-rowing performance in AB individuals, several limitations should be considered. First, as we intended to test the feasibility of the coaching system, we only had a small sample of AB in this study. Therefore, the results should not be generalized to other groups, and we need a larger-scale study to make a conclusion on the system’s applicability to various individuals, including those with SCI. Second, the rowing experience of our study sample was relatively broad, including an individual with no prior rowing experiment and one varsity athlete. Lastly, the duration of our FES-rowing training with the coaching system was relatively short, even though it resulted in improved performance. Future studies shall investigate the validation of this coaching system in other populations to determine possible therapeutic effects and any accessibility modifications that may be required for these individuals.

## 5. Conclusions

Our results demonstrated that VL activation during rowing began consistently before the individuals reached the anterior-most seat position. Therefore, to guide the timing of the manual button-pressing for administering FES, seat position at the onset of VL activation was used. Concerning the coaching system, it provides a safe method of improving FES-rowing performance in AB individuals, as demonstrated by increased power and work outputs during the coaching condition, indicating faster performance and longer distance traveled per stroke.

## Data Availability

Data available on request from the corresponding author.
